# Impact of Microvascular Complication Burden on the Risk of Dementia and Cognitive Decline in Patients With Diabetes: A Systematic Review of Latest Evidence

**DOI:** 10.7759/cureus.109445

**Published:** 2026-05-22

**Authors:** Sania Akhtar Gul, Jennifer John, Reyna Rehan, Mohamad Faisal Bahboh, Nazia Akhtar, Syed Ali Hussain Shah Kazmi, Haroon Javed, Saheel Ahmed, Fatima Ali, Abdelhamid Abdelgayoum Suliman Bashir, Muhammad Salman Arif

**Affiliations:** 1 Medicine, Gulf Medical University, Ajman, ARE; 2 Internal Medicine, Ras Al Khaimah Medical and Health Sciences University, Sharjah, ARE; 3 Internal Medicine, Sultanah Aminah Hospital, Johor, MYS; 4 Surgery, Ras al-Khaimah Medical and Health Sciences University, RAK, ARE; 5 Internal Medicine, Shifa College of Medicine, Islamabad, PAK; 6 Medicine, Shifa International Hospital, Islamabad, PAK; 7 Medicine, Georgian National University, Tbilisi, GEO; 8 Medicine, Khyber Medical College, Peshawar, PAK; 9 Surgery, Almanagil Teaching Hospital, Almanagil, SDN

**Keywords:** cognitive decline, dementia, diabete mellitus, microvascular complications, types 2 diabetes

## Abstract

Diabetes mellitus is an established risk factor for dementia; however, the specific contributions of microvascular complications and glycemic control to cognitive decline remain unclear. This systematic review synthesizes current evidence on the association between microvascular complication burden and dementia risk in individuals with diabetes. A comprehensive search of PubMed, the Cochrane Library, and Embase was conducted for studies published through March 2026, including cohort and cross-sectional studies evaluating microvascular complications or glycemic control in relation to dementia or cognitive decline. The Newcastle-Ottawa Scale was used to evaluate the quality of the study. A total of 1,697,156 participants from five studies, two cross-sectional and three cohort, were included. Cohort studies consistently showed that microvascular problems are linked to a higher incidence of dementia; the largest relationships were found with diabetic neuropathy (hazard ratio (HR) 1.12-1.26) and nephropathy (HR 1.13-1.23), but results for retinopathy were inconsistent. Furthermore, an 8% increased risk of dementia was associated with every 1% increase in HbA1c, and cognitive decline was independently influenced by HbA1c variability. Additionally, significant associations were found between the severity of microvascular problems and HbA1c levels (*r* = 0.839-0.870). In individuals with diabetes, microvascular complications-especially neuropathy and nephropathy-are generally linked to a higher risk of dementia, and poor glycemic management seems to worsen both microvascular damage and cognitive decline. These results emphasize the importance of optimizing glycemic management and routine screening for microvascular complications as viable methods to maintain cognitive health.

## Introduction and background

Diabetes mellitus, a significant global public health concern, is characterized by persistent hyperglycemia and increasing metabolic dysfunction. Macrovascular and microvascular sequelae are the two main categories of its long-term problems. The latter includes diabetes retinopathy, neuropathy, and nephropathy, which are mostly caused by chronic small artery damage brought on by prolonged hyperglycemia [1]. Numerous organ systems are impacted by these microvascular changes, which gradually impair function and lower quality of life [2]. A significant percentage of people with diabetes experience at least one microvascular consequence; this is especially true for those with poor glycemic control, longer illness duration, or poor treatment adherence. Significantly, longer-term diabetes has been repeatedly linked to more severe microvascular damage as well as an elevated risk of dementia and cognitive decline, most likely as a result of cumulative vascular, inflammatory, and metabolic insults over time. Heterogeneity in the observed relationships between microvascular problems and cognitive outcomes may be partially explained by the fact that diabetes duration varied significantly among the included studies and was not consistently recorded. Global healthcare demands are rising, life expectancy is declining, and morbidity is rising due to the cumulative impact of these issues [3].

Recent evidence indicates that diabetes can directly affect the brain through microvascular dysfunction, extending beyond its traditionally recognized end-organ complications. Individuals with diabetes have an approximate 50% higher risk of the development of cognitive impairment and dementia compared to those without the disease [4]. Chronic hyperglycemia plays an important role in this process, alongside underlying mechanisms such as oxidative stress, low-grade inflammation, and endothelial dysfunction, all of which progressively disrupt cerebral microcirculation [5]. These alterations alter the cerebral blood flow and compromise the integrity of the blood-brain barrier, promoting small vessel disease and ultimately contributing to the development of cognitive decline and dementia [6].

In this review, microvascular complication burden refers to the presence, number, and co-occurrence of diabetic microvascular complications within an individual, ranging from a single complication (e.g., isolated neuropathy) to multiple concurrent complications (e.g., neuropathy with nephropathy and/or retinopathy). There is currently no widely recognized composite burden index, which adds to the literature's heterogeneity. Some studies also use severity-based classifications. Crucially, this review distinguishes between dementia (a clinical illness marked by considerable functional impairment) and cognitive decline (a spectrum of measurable impairment across cognitive domains), since these outcomes vary in severity, diagnostic criteria, and clinical consequences [5,7].

Hence, there are still significant gaps in the body of knowledge despite the growing amount of research in this field. Prior research has mostly focused on individual microvascular problems, with little assessment of their combined burden (e.g., co-occurrence or cumulative number of issues) in relation to cognitive outcomes. Furthermore, important variables, including the length of diabetes, glycemic variability, and cardiovascular comorbidities, which may have a substantial impact on the severity of microvascular disease and the risk of dementia, are not consistently adjusted for. Furthermore, there is uncertainty in the interpretation of results because previous investigations have not reliably distinguished between clinically diagnosed dementia and cognitive decline. Despite possible variations in vascular and neurodegenerative pathways, there is also a lack of synthesis among dementia subgroups [7].

Therefore, this systematic review was conducted to address these gaps by comprehensively evaluating both individual and cumulative microvascular complication burden in relation to cognitive decline and dementia outcomes, while also distinguishing between outcome definitions and dementia subtypes. This approach aims to provide a more structured and clinically interpretable synthesis of the current evidence base.

## Review

Methodology

Our systematic review and meta-analysis were conducted in accordance with the Preferred Reporting Items for Systematic Reviews and Meta-Analyses (PRISMA) guidelines (CRD420261373452) [8].

Study selection process and search strategy 

We developed a comprehensive search strategy to examine the association between microvascular complications and dementia and cognitive decline in patients with diabetes mellitus. A systematic search was conducted across PubMed, the Cochrane Library, Embase, and Scopus to identify relevant studies. The search focused on the MeSH terms “microvascular complications,” including “retinopathy,” “neuropathy,” “nephropathy,” “hypoglycemia,” and “glycemic control,” and their relationship with cognitive outcomes. Keywords and their synonyms included “diabetes mellitus,” “type 2 diabetes,” “microvascular complications,” “HbA1c,” and “cognitive decline.” Full search strategies are provided in the Appendix.

A structured and systematic approach was used to ensure comprehensive and reproducible study selection. Primary screening was performed by two reviewers using titles and abstracts, and discrepancies were resolved through a third review. Secondary screening was conducted using full-text articles. Studies meeting the predefined inclusion criteria after full-text assessment were included in the systematic review.

Eligibility criteria

We included cohort and cross-sectional studies that reported microvascular complications in patients with DM, included exposure data, and were published between 2000 and March 2026. We excluded studies involving non-diabetic patients, non-peer-reviewed studies, studies without full text, and studies not available in English. Moreover, opinions, reviews, case reports, abstracts, and similar publication types were excluded.

Data extraction

Data from included studies were systematically extracted using a standardized template (Excel sheet) to ensure consistency and completeness. Key variables recorded included author details, study design, participant characteristics, exposure, comparator group, and reported outcomes. Additionally, sample size, type of microvascular complications, and baseline characteristics were also documented. The primary outcomes of interest were Alzheimer disease, cognitive decline, vascular dementia, and relevant glycemic control indicators reported within each study.

Quality assessment

A comprehensive quality assessment was conducted for all included studies using appraisal-validated tools appropriate to the study design. Cohort studies were evaluated using the Newcastle-Ottawa Quality Assessment Scale, which assesses three domains: selection (4 items), comparability (1 item), and outcome (3 items), with a maximum score of 9 stars [9]. Cross-sectional studies were assessed using an adapted Newcastle-Ottawa Scale, encompassing seven domains: representativeness of the sample, sample size justification, non-response rate, ascertainment of exposure or screening tool, control of confounding factors, outcome assessment, and appropriate statistical analyses, with a maximum score of 9. Based on these criteria, studies were categorized as having low, moderate, or high risk of bias. Detailed summary tables (Tables 1-2) were generated to present the quality assessment across all domains for each included study [9].

**Table 1:** Newcastle-Ottawa Quality assessment scale for cohort studies. ☆: Indicates adherence to the criteria. Numbers in parentheses () represent the maximum possible score for each criterion. The total score reflects the overall quality assessment of each study. A maximum score of 9 indicates high quality, a score of 6 or above indicates good quality, and a score below 6 indicates low quality. Included studies [10-12].

Study ID	Representativeness of the exposed cohort (1)	Selection of the non-exposed cohort (1)	Ascertainment of exposure (1)	Demonstration that the outcome of interest was not present at the start of the study (1)	Comparability of cohorts based on the design or analysis controlled for confounders (2)	Assessment of outcomes (1)	Was the follow-up long enough for outcomes to occur (1)	Adequacy of follow-up of cohorts (1)	Total score (9)
Yen et al. (2024)	☆	☆	☆	☆	☆☆	☆	☆	☆	9/9
Zheng et al. (2021)	☆	☆	☆	☆	☆☆	☆	☆	☆	9/9
Liu et al. (2024)	☆	☆	☆	☆	☆☆	☆	☆	☆	9/9

**Table 2:** Newcastle-Ottawa quality assessment scale (adapted for cross-sectional studies). ☆: Indicates adherence to the criteria. Numbers in parentheses ( ) represent the maximum possible score for each criterion. The total score reflects the overall quality assessment of each study. A maximum score of 9 indicates high quality; scores of 6 and above indicate good quality; scores below 6 indicate low quality. Includes studies [13,14].

Study	Representativeness of the cases (1)	Sample size justification (1)	Non-response rate (1)	Ascertainment of screening/surveillance tool (2)	Investigation of confounders (1)	Assessment of outcomes (2)	Statistical test appropriateness (1)	Total score (9)
Memon et al. (2025)	-	-	-	☆☆	-	☆☆	☆	5/9
Ba-Tin et al. (2011)	-	☆	-	☆☆	☆	☆☆	☆	7/9

Results

**Figure 1:**
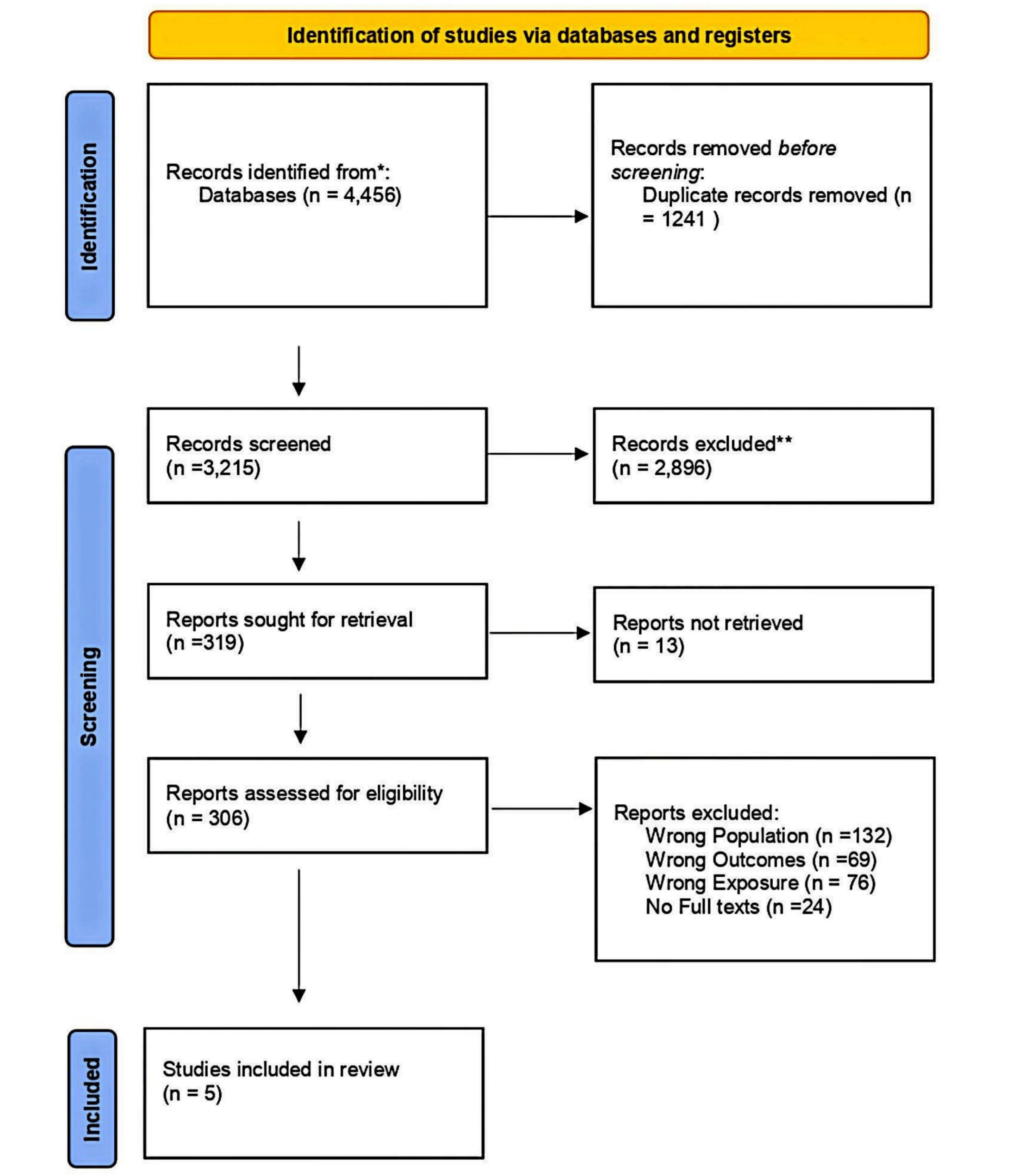
PRISMA flow diagram of study selection. This figure illustrates the study selection process according to PRISMA guidelines, including the identification, screening, eligibility, and inclusion stages. PRISMA, Preferred Reporting Items for Systematic Reviews and Meta-Analyses

Starting with the databases, the initial search identified 4,456 studies. Duplicates - exactly 1,241 - were removed early. Screening then focused on titles and abstracts across 3,215 records. Most did not meet the criteria; 2,896 were excluded. From the remaining studies, we attempted to retrieve 319 full texts. Thirteen, however, were not accessible. Of the 306 reports assessed, five ultimately qualified for the final analysis. Most were excluded: 132 involved incorrect populations, 76 had improper exposures, 69 failed outcome measures, and 24 had no accessible full text. As a result, only studies meeting all criteria were included in the review (Figure 1).

Study characteristics

Five original research studies [10-14] met the inclusion criteria for this systematic review: three large-scale cohort studies [10-12] and two cross-sectional studies [13,14], with a combined sample of 1,697,156 participants. Yen et al. [10] from Taiwan examined 1,212,966 patients with type 2 diabetes from the National Health Insurance Research Database, with a mean follow-up of 6.5 years. The cohort studies included Zheng et al. [11] from the United Kingdom, who analyzed 457,902 patients with type 2 diabetes from the Clinical Practice Research Datalink over a median follow-up of six years, and Liu et al. [12] from the United Kingdom, who investigated 26,173 participants with type 2 diabetes from the UK Biobank over a median follow-up of 11.5 years. Memon et al. [13] from Pakistan included 30 patients with type 1 diabetes mellitus. The two cross-sectional studies included Ba-Tin et al. [14] from the United Kingdom, which enrolled 75 participants, including healthy controls and patients with diabetes with and without microvascular complications. Study characteristics are summarized in Table 3.

**Table 3:** Study characteristics, patients’ demographics, and baseline characteristics. Included studies [10-14]. AD, Alzheimer’s disease, BMI, body mass index, CANTAB, Cambridge Neuropsychological Test Automated Batteries, CKD, chronic kidney disease, CPRD, Clinical Practice Research Datalink, D, diabetes without complications, DC, diabetes with complications, DKD, diabetic kidney disease, DN, diabetic neuropathy, DR, diabetic retinopathy, eGFR, estimated glomerular filtration rate, ETDRS, Early Treatment Diabetic Retinopathy Study, HbA1c, glycated hemoglobin, HES, Hospital Episode Statistics, ICD, International Classification of Diseases, IQR, interquartile range, KDIGO, Kidney Disease: Improving Global Outcomes, MVD, microvascular disease, NHIRD, National Health Insurance Research Database, NR, not reported, ONS, Office for National Statistics, SD, standard deviation, T1DM, type 1 diabetes mellitus, T2DM, type 2 diabetes mellitus, TCNS, Toronto Clinical Neuropathy Score, VaD, vascular dementia; aHR, adjusted hazard ratio

Study	Country	Design	Sample size (*n*)	Population	Age (years)	Male (%)	Follow-up duration	Diabetes Duration (years)	Baseline hba1c (%)	BMI (kg/m²)	Current smoker (%)	Hypertension (%)	MVD definition	Outcome assessment
Zheng et al. (2021) [11]	United Kingdom	Cohort	457,902	T2DM	Mean 64.5 (SD 10.8)	52.1	Median 6 years (0-31)	Mean 0.6	Mean 7.4 (SD 2.1)	≥30: 42.3%	19.0	85.0	Clinical codes (CPRD) for retinopathy, neuropathy, nephropathy, hypoglycemia	Incident all-cause dementia via CPRD, HES, ONS
Yen et al. (2024) [10]	Taiwan	Cohort	1,212,966	T2DM	≥50 (matched)	53.3 (matched)	Mean 6.5 years	NR	NR	≥30: 45.1% (matched)	17.9 (matched)	92.0 (matched)	ICD-9/10 codes for DKD, DR, DN within 1 year of diagnosis	AD, VaD, all-cause dementia via NHIRD
Liu et al. (2024) [12]	United Kingdom	Cohort	26,173	T2DM	40-69 (baseline)	59.2	Median 11.5 years	Median 3.0 (IQR 0-8)	Median 6.8 (IQR 6.1-7.8)	Mean 30.9 (SD 5.5)	11.0	73.1	Self-report + hospital records (retinopathy, neuropathy); CKD via eGFR <60 or albuminuria	Dementia subtypes (AD, VaD) via hospital records
Ba-Tin et al. (2011) [14]	United Kingdom	Cross-sectional	75	T1DM and T2DM	Controls: 53 (41-68); D: 60 (45-80); DC: 61 (42-80)	Controls: 44%; D: 61%; DC: 52%	N/A	D: 7.4 (1-27); DC: 20.8 (1-46)	D: 7.73; DC: 7.53	D: 30; DC: 32	0 (all groups)	D: 30%; DC: 81%	Clinical diagnosis (retinopathy, neuropathy, nephropathy)	Cognitive function (CANTAB, 7 domains)
Memon et al. (2025) [13]	Pakistan	Cross-sectional	30	T1DM	Mean 24.2 (SD 3.5)	53.3	N/A	Mean 6.3 (SD 1.5)	Mean 7.65 (SD 1.15)	NR	NR	NR	KDIGO (nephropathy), ETDRS (retinopathy), TCNS (neuropathy)	Microvascular complication severity

Outcomes

The results and outcomes are summarized in Table 4.

**Table 4:** Summary of included studies evaluating microvascular complications, dementia, and cognitive outcomes in diabetes. Included studies [10-14]. AD, Alzheimer’s disease, BMI, body mass index, CANTAB, Cambridge Neuropsychological Test Automated Batteries, CKD, chronic kidney disease, CPRD, Clinical Practice Research Datalink, D, diabetes without complications, DC, diabetes with complications, DKD, diabetic kidney disease, DN, diabetic neuropathy, DR, diabetic retinopathy, eGFR, estimated glomerular filtration rate, ETDRS, Early Treatment Diabetic Retinopathy Study, HbA1c, glycated hemoglobin, HES, Hospital Episode Statistics, ICD, International Classification of Diseases, IQR, interquartile range, KDIGO, Kidney Disease: Improving Global Outcomes, MVD, microvascular disease, NHIRD, National Health Insurance Research Database, NR, not reported, ONS, Office for National Statistics, SD, standard deviation, T1DM, type 1 diabetes mellitus, T2DM, type 2 diabetes mellitus, TCNS, Toronto Clinical Neuropathy Score, VaD, vascular dementia; HR, hazard ratio

Author (year)	Study design	Population	Exposure	Outcomes	Results
Zheng et al. (2021) [11]	Retrospective cohort	457,902 patients with T2DM aged ≥50 years (UK CPRD); median follow-up six years	Presence of microvascular complications (retinopathy, neuropathy, nephropathy, hypoglycemia); HbA1c levels and variability	All-cause dementia	Any MVD: HR 1.10 (1.06-1.14); nephropathy: HR 1.23 (1.13-1.33); neuropathy: HR 1.25 (1.18-1.33); retinopathy: HR 1.07 (1.03-1.11); hypoglycemia: HR 1.30 (1.22-1.39); per 1% ↑ HbA1c: HR 1.08 (1.07-1.09); HbA1c ≥10%: HR 1.40 (1.32-1.49); HbA1c variability (per 1 SD): HR 1.03 (1.01-1.04)
Yen et al. (2024) [10]	Retrospective cohort	1,212,966 patients with T2DM aged ≥50 years (Taiwan NHIRD); mean follow-up 6.5 years	Early microvascular disease (DKD, DR, DN within 1 year of diagnosis)	AD, VaD, all-cause dementia	Any MVD → All-cause: aHR 1.13 (1.09-1.17); AD: aHR 1.15 (1.07-1.24); VaD: aHR 1.16 (1.08-1.25); DKD → All-cause: aHR 1.13 (1.06-1.20); AD: aHR 1.16 (1.02-1.32); VaD: aHR 1.21 (1.06-1.38); DN → All-cause: aHR 1.12 (1.06-1.17); AD: aHR 1.14 (1.03-1.27); VaD: aHR 1.14 (1.02-1.28); DR → All-cause: aHR 0.97 (0.86-1.11), NS
Liu et al. (2024) [12]	Prospective cohort	26,173 participants with T2DM aged 40-69 years (UK Biobank); median follow-up 11.5 years	Number of microvascular diseases (retinopathy, peripheral neuropathy, CKD)	Stroke, AD, VaD, all-cause dementia	1 MVD: HR 1.37 (1.18-1.60); 2 MVDs: HR 1.73 (1.34-2.23); 3 MVDs: HR 4.28 (2.33-7.86); retinopathy: HR 1.38 (1.19-1.61); PN: HR 1.26 (1.06-1.49); CKD: HR 1.21 (1.04-1.42); 3 MVDs → AD: HR 6.96 (3.02-16.01); retinopathy → AD: HR 1.61 (1.25-2.06); PN → AD: HR 1.37 (1.03-1.82); 3 MVDs → VaD: HR 3.81 (1.40-10.42); PN → VaD: HR 1.52 (1.12-2.06); CKD → VaD: HR 1.37 (1.05-1.79)
Ba-Tin et al. (2011) [14]	Cross-sectional	75 participants: 25 controls (23 diabetics without MVD (27 diabetics with MVD	Presence of peripheral MVD (retinopathy, neuropathy, nephropathy)	Cognitive function (CANTAB tests)	Diabetics performed worse vs. controls: reaction time (*P* = 0.003), rapid visual processing (*P* = 0.001); No difference between diabetics with vs. without MVD across domains: reaction time (*P* = 0.624), rapid visual processing (*P* = 0.576), pattern recognition (*P* = 0.86), spatial recognition (*P* = 0.86), paired associate learning (*P* = 0.63), match to sample (*P* = 0.89), motor skills (*P* = 0.43)
Memon et al. (2025) [13]	Cross-sectional	30 patients with T1DM; mean age 24.2 years; duration 6.3 years	HbA1c levels; severity of complications (KDIGO, ETDRS, TCNS)	Severity of MVD	HbA1c correlation: Retinopathy *r* = 0.864 (*P* < 0.001); neuropathy *r* = 0.870 (*P* < 0.001); nephropathy *r* = 0.839 (*P* < 0.001); mean HbA1c: No retinopathy 6.43%, mild NPDR 7.20%, moderate NPDR 8.16%; Neuropathy: 6.59% (mild) → 9.40% (severe); ANOVA *P* < 0.001

Primary outcomes 

Composite Microvascular Complications and All-Cause Dementia

All three cohort studies [10-12] consistently demonstrated that the presence of any microvascular complication increases the risk of all-cause dementia in patients with diabetes, although effect sizes varied. Yen et al. [10], analyzing 1,212,966 patients over a mean follow-up of 6.5 years, found a 13% increased risk (adjusted hazard ratio (aHR) 1.13; 95% CI 1.09-1.17; *P* < 0.001) after propensity score matching. Moreover, Zheng et al. [11], in a cohort of 457,902 patients with type 2 diabetes followed for a median of six years, reported a 10% increased risk of incident dementia (HR 1.10; 95% CI 1.06-1.14; *P* < 0.05) after adjusting for demographic factors, comorbidities, diabetes duration, medications, and HbA1c. A two-year lag period was applied to reduce reverse causality. The cumulative incidence of dementia remained significantly higher in patients with microvascular disease (log-rank *P* < 0.001). Liu et al. [12], in 26,173 participants followed for 11.5 years, demonstrated a clear dose-response relationship: HR 1.37 (one complication), 1.73 (two complications), and 4.28 (three complications), all *P* < 0.001, confirming that cumulative microvascular burden substantially increases dementia risk.

Diabetic Kidney Disease and All-Cause Dementia

All three cohort studies [10-12] showed that diabetic kidney disease is independently associated with increased dementia risk. Yen et al. [10] found a 13% increase (aHR 1.13; 95% CI 1.06-1.20; *P* < 0.001), with a focus on early-stage disease. Zheng et al. [11] reported a 23% increased risk (HR 1.23; 95% CI 1.13-1.33; *P* < 0.05). Liu et al. [12] reported a 21% increase (HR 1.21; 95% CI 1.04-1.42; *P* = 0.014), independent of other complications. These findings suggest that kidney disease contributes independently to dementia risk across populations.

Diabetic Neuropathy and All-Cause Dementia

Neuropathy showed one of the strongest and most consistent associations with dementia among all three cohort studies [10-12]. Zheng et al. [11] reported HR 1.25 (95% CI 1.18-1.33; *P* < 0.05). Yen et al. [10] reported aHR 1.12 (95% CI 1.06-1.17; *P* < 0.001). Liu et al. [12] reported HR 1.26 (95% CI 1.06-1.49; *P* = 0.009). The consistency across studies highlights neuropathy as a robust predictor of cognitive decline.

Diabetic Retinopathy and All-Cause Dementia

Results across the three cohort studies [10-12] showed inconsistent associations with retinopathy. Contrary findings were reported by Yen et al. [10], in which adjusted results showed no association (aHR 0.97; 95% CI 0.86-1.11; *P* = 0.69). However, a significant increase in risk was reported by Zheng et al. [11], with an HR of 1.07 (95% CI 1.03-1.11; *P* < 0.05). In contrast, Liu et al. [12] observed a notably higher HR (HR 1.38; 95% CI 1.19-1.61; *P* < 0.001). Differences in populations, disease stages, or outcome assessment methods may explain these divergent patterns.

Microvascular Complications and Alzheimer’s Disease

Regarding Alzheimer’s disease outcomes, two studies [10,12] reported variations in associations between individual microvascular complications and risk estimates. Yen et al. [10] demonstrated a statistically significant 15% increased risk of Alzheimer’s disease associated with any microvascular complication (aHR 1.15; 95% CI 1.07-1.24; *P* < 0.001). At the organ-specific level, diabetic nephropathy (HR 1.16; *P* = 0.02) and neuropathy (HR 1.14; *P* = 0.01) were both significantly associated with increased risk, whereas retinopathy was not (HR 1.02; *P* = 0.88). In contrast, Liu et al. [12] observed a pronounced dose-response relationship, where the presence of three concurrent microvascular complications was associated with a substantially elevated risk of Alzheimer’s disease (HR 6.96; 95% CI 3.02-16.01; *P* < 0.001). In this study, retinopathy (HR 1.61; *P* < 0.001) and neuropathy (HR 1.37; *P* = 0.03) remained significantly associated with Alzheimer’s disease, whereas nephropathy was not statistically significant (HR 1.16; *P* = 0.25). Despite inter-study variability in the contribution of individual complications, neuropathy demonstrated the most consistent association with Alzheimer’s disease across both studies.

Microvascular Complications and Vascular Dementia

For vascular dementia outcomes, two studies [10,12] demonstrated broadly consistent associations between diabetic microvascular complications and increased risk. Yen et al. [10] reported a 16% increased risk of vascular dementia associated with any microvascular complication (aHR 1.16; 95% CI 1.08-1.25; *P* < 0.001). At the individual-complication level, diabetic nephropathy (HR 1.21; *P* = 0.004) and neuropathy (HR 1.14; *P* = 0.02) were both significantly associated with increased risk, whereas retinopathy was not (HR 1.05; *P* = 0.73). Similarly, Liu et al. [12] reported that the presence of three concurrent microvascular complications was associated with a substantially elevated risk of vascular dementia (HR 3.81; 95% CI 1.40-10.42; *P* = 0.009). In this cohort, neuropathy (HR 1.52; *P* = 0.008) and nephropathy (HR 1.37; *P* = 0.02) remained significantly associated with vascular dementia, while retinopathy showed a non-significant association (HR 1.25; *P* = 0.10). Overall, findings across both studies were more consistent for vascular dementia than for Alzheimer’s disease, particularly with respect to neuropathy and nephropathy as contributing risk factors.

Secondary outcomes

Glycemic Control and Dementia Risk

Glycemic control demonstrated a significant, multifaceted relationship. Zheng et al. [11] found a consistent dose-response association, with each 1% increase in HbA1c associated with an 8% higher risk of dementia (HR 1.08; 95% CI 1.07-1.09; *P* < 0.05). Dementia risk rose gradually in comparison to the reference group (6%-7%), with lower risk at HbA1c <6% (HR 0.86; 95% CI 0.83-0.89), higher risk at 8%-9% (HR 1.15; 95% CI 1.09-1.21), 9%-10% (HR 1.26; 95% CI 1.17-1.34), and ≥10% (HR 1.40; 95% CI 1.32-1.49). Memon et al. [13] found strong positive correlations between HbA1c and the severity of diabetic complications, such as nephropathy (*r* = 0.839), retinopathy (*r* = 0.864), and neuropathy (*r* = 0.870), all of which were statistically significant (*P* < 0.001), providing additional evidence for the connection between glycemic dysregulation and microvascular damage. A substantial correlation between HbA1c levels and the severity of complications was further confirmed by analysis of variance (*P* < 0.001), indicating a close relationship between poor glycemic control and progressive microvascular damage. Ba-Tin et al. [14], on the other hand, found no statistically significant difference in HbA1c levels between patients with and without microvascular complications (7.53% vs. 7.73%; *P* = 0.97). This suggests that factors other than glycemic control may impact the development of complications.

Cognitive Function and Microvascular Complications

Ba-Tin et al. [14] assessed cognition using the Cambridge Neuropsychological Test Automated Batteries. Diabetic patients performed worse than controls in reaction time (0.515 vs. -0.354 vs. -0.525; *P* = 0.003) and rapid visual information processing (0.025 vs. -1.037 vs. -1.007; *P* = 0.001). However, no significant differences were observed between diabetic patients with and without complications (reaction time: *P* = 0.624; processing: *P* = 0.576). In contrast, Yen et al. [10], Zheng et al. [11], and Liu et al. [12] provided indirect evidence via dementia incidence, consistently showing that poor glycemic control and microvascular complications increase the risk of progression to dementia.

Discussion

Summary of Results

This systematic review evaluated the microvascular complications and glycemic control with dementia and cognitive decline in patients with diabetes mellitus. The primary outcomes include incidence of dementia, Alzheimer’s disease, vascular dementia and measures of cognitive function. Liu et al. [12] showed that a progressive increase in dementia risk with a higher number of microvascular complications, reaching an HR of 4.28, among patients with three concurrent complications. The association between diabetic retinopathy and dementia risk showed variability, with two studies reporting a significant association while one found no significant link. Regarding glycemic control, HbA1c level and greater than HbA1c variability were consistently associated with an increased risk of dementia, alongside a strong correlation between HbA1c and the severity of microvascular complications. However, findings from cross-sectional studies indicated that although diabetes was associated with cognitive impairment, the presence of microvascular complications did not significantly differentiate cognitive performance among diabetic individuals.

Comparison With Existing Literature

The results of this comprehensive review support and expand upon earlier epidemiological data relating diabetes to cognitive decline. Previous meta-analyses of cohort studies have demonstrated a strong link between diabetes and dementia. In a meta-analysis of 14 prospective observational studies, Gudala et al. [15] discovered that people with diabetes were 53% more likely to develop dementia than those without the disease. Similarly, Chatterjee et al. [16] reported a 62% increased risk of dementia in women with diabetes, and Zhang et al. [17] observed a 47% increased risk of Alzheimer’s disease in people with diabetes. By focusing on the distinct roles of different microvascular complications rather than diabetes itself, the current analysis provides a more detailed insight.

Previous research supports the consistent link between dementia and diabetic neuropathy found in this review. In a study of 1,642 participants, Hicks et al. [18] reported an association between dementia and mild cognitive impairment with peripheral neuropathy, characterized by monofilament insensitivity. Peripheral neuropathy was also linked to lower cognitive performance in older populations. Studies suggesting that neuropathy and cognitive impairment share pathogenic pathways, such as inflammation, oxidative stress, and persistent hyperglycemia, provide biological plausibility for this association [19].

Furthermore, the association between diabetic kidney disease and dementia found in this review is consistent with previous research. In a systematic review and meta-analysis of 22 prospective studies, Deckers et al. [20] reported that chronic renal disease was associated with a 25% higher incidence of dementia and cognitive impairment. In particular, diabetic kidney disease was linked to a 15% higher incidence of dementia in individuals with type 2 diabetes [21]. The accumulation of uremic toxins, persistent inflammation, and shared vascular risk factors are possible explanations for this association [21].

Diabetes-related microvascular issues seem to be linked to dementia and cognitive decline as well as more general neuropsychiatric consequences, such as depression, indicating a common underlying pathway of systemic microvascular and neurovascular dysfunction. Nephropathy, retinopathy, and neuropathy may all contribute differentially to brain-related outcomes, according to evidence from earlier systematic reviews, with nephropathy demonstrating particularly substantial relationships with cognitive decline. However, the need for standardized longitudinal research to elucidate these connections is highlighted by variation in study populations, outcome definitions, and methods for confounding correction, which limits direct comparability [2].

Moreover, the differing results among the included studies on diabetic retinopathy are indicative of the broader body of research on this subject. A comprehensive analysis of the relationship between diabetic retinopathy and cognitive impairment was conducted by Crosby-Nwaobi et al. [22]. The results were mixed, with some studies revealing substantial relationships and others not. Similarly, it was pointed out that although dementia and retinopathy share mechanisms, including oxidative stress, inflammation, and vascular damage, the epidemiological evidence is still inconsistent. However, diabetic retinopathy was associated with a 21% increased risk of all-cause dementia, but subgroup analyses revealed significant heterogeneity across studies. This variation may be attributed to differences in retinopathy severity, duration, population characteristics, and healthcare systems [22].

In a population-level cohort analysis of 49,027 patients with type 2 diabetes, Brownrigg et al. [23] found that the number of microvascular complications gradually increased the risk of cardiovascular events. This idea is extended to cognitive outcomes in the current review, which proposes that the overall burden of microvascular illness is a crucial factor in dementia risk. The results about glycemic control are consistent with earlier studies. Similarly, in the Atherosclerosis Risk in Communities study, Rawlings et al. [24] found that elevated HbA1c levels were linked to a higher risk of dementia and mild cognitive impairment. Li et al. [25], who showed that visit-to-visit changes in fasting plasma glucose and HbA1c were linked with higher Alzheimer's disease risk in the Taiwan Diabetes Study, corroborate the conclusion that HbA1c variability is associated with dementia risk. Moreover, the ACCORD-MIND trial, a large randomized controlled study, evaluated whether intensive glycemic control could improve cognitive outcomes in patients with type 2 diabetes but did not demonstrate a clear cognitive benefit despite improved glycemic parameters. These findings suggest that tighter glucose control alone may not be sufficient to modify cognitive trajectories once vascular and microvascular damage is established. This highlights that dementia risk in diabetes is likely driven by complex structural and microvascular changes rather than glycemic levels alone. [26]

Hence, the disparity between the cohort study results and the cross-sectional cognitive function study is consistent with earlier findings. Glycemic management was associated with 9-year cognitive deterioration, whereas cross-sectional relationships at baseline were weaker, according to Yaffe et al. [27]. While longitudinal studies more reliably show cognitive impairment with time, cross-sectional investigations of cognitive function in diabetes sometimes produce conflicting results. This implies that rather than producing cross-sectional disparities at a single time point, microvascular problems may hasten the course of cognitive deterioration.

Strengths, Limitations, and Future Research

A thorough literature search across numerous databases, a rigorous quality assessment using the Newcastle-Ottawa Scale, and an assessment of both composite and individual microvascular problems across dementia subtypes are some of the systematic review's strong points. Significant population-level data on the relationship between diabetic microvascular illness and cognitive outcomes were provided by three included cohort studies, comprising over 1.6 million individuals. However, it is crucial to recognize several significant limitations. Only five studies met the eligibility criteria after screening more than 4,000 records, underscoring the dearth of high-quality data specifically examining this link. Additionally, the robustness and generalizability of the results are limited because two of the included investigations were cross-sectional studies with comparatively small sample sizes and limited correction for confounding variables. There was also significant variation among studies in definitions of microvascular complications, cognitive evaluation methodologies, study populations, follow-up duration, and correction for diabetes-related characteristics, such as disease duration and glycemic control. Furthermore, diabetes duration was inconsistently recorded, while being a significant predictor of both microvascular problem severity and dementia risk. The prevalence of European and East Asian people may limit the generalizability of the findings to other ethnic and geographical groups. As a result, the conclusions of this analysis should be regarded with caution, given that the present database is small and observational. Future research should concentrate on large-scale prospective longitudinal studies with standardized definitions of microvascular complications, repeated cognitive assessments, detailed adjustments for diabetes duration and glycemic control, and inclusion of more diverse populations to better understand the temporal and biological relationship between diabetic microvascular disease and dementia.

Future Recommendations

Future research should focus on large prospective cohort studies that assess the independent contribution of individual microvascular complications, particularly diabetic nephropathy and neuropathy, to various dementia subtypes after controlling for major confounders such as diabetes duration, HbA1c variability, hypertension, and cardiovascular disease. Standardized diagnostic criteria for microvascular problems and cognitive impairment are required to increase study comparability. Furthermore, longitudinal investigations that include serial cognitive testing and neuroimaging biomarkers may help determine whether progressive microvascular injury directly contributes to neurodegeneration or reflects systemic vascular disease load. Given the current database's inadequate representation of non-European people, future multicenter research should include diabetic patients from diverse ethnic and geographic backgrounds to improve external validity. Randomized interventional studies to determine if stringent glycemic control or early therapy of microvascular problems would minimize the incidence of long-term dementia would also provide valuable clinical insight into future preventative therapies.

## Conclusions

Our systematic study found a link between diabetic microvascular problems, particularly diabetic neuropathy and nephropathy, and an increased risk of dementia in people with diabetes. Higher HbA1c levels and variability are also linked to microvascular disease load and cognitive impairment, according to the research analyzed. However, these findings should be regarded with caution because the current data are limited, diverse, and mostly observational. The conclusions are limited, and causal inference is not possible due to the small number of eligible studies, uneven confounding variable correction, inconsistent study methodology, and inclusion of tiny cross-sectional studies. Crucially, there is currently no proof that glycemic optimization or the avoidance of microvascular problems actually lowers the risk of dementia, even though poor glycemic control seems to be associated with poor cognitive results. Thus, more extensive prospective longitudinal research and controlled trials are needed to elucidate the temporal association, underlying mechanisms, and possible therapeutic consequences between dementia and diabetic microvascular illness.

## Appendices

Appendix 

**Table 5:** Supplementary Materials

Pubmed =1319	Embase=2234	Scopus= 789	Cochrane CENTRAL =114
(“Diabetes Mellitus”[MeSH Terms] OR “diabet*”[Title/Abstract]) AND (“Diabetic Retinopathy”[MeSH Terms] OR “Diabetic Nephropathies”[MeSH Terms] OR “Diabetic Neuropathies”[MeSH Terms] OR “microvascular complication*”[Title/Abstract] OR “microvascular disease*”[Title/Abstract] OR “retinopath*”[Title/Abstract] OR “nephropath*”[Title/Abstract] OR “neuropath*”[Title/Abstract]) AND (“Dementia”[MeSH Terms] OR “Alzheimer Disease”[MeSH Terms] OR ”dement*”[Title/Abstract] OR “cognitive decline”[Title/Abstract] OR “cognitive impairment”[Title/Abstract] OR “cognitive dysfunction”[Title/Abstract])	(‘diabetes mellitus’/exp OR ‘type 1 diabetes mellitus’/exp OR ‘type 2 diabetes mellitus’/exp OR diabet*:ti,ab) AN” (‘diabetic retinopathy’/exp OR ‘diabetic nephropathy’/exp OR ‘diabetic neuropathy’/exp OR ‘microangiopathy’/exp OR ((microvascular NEXT/1 (complication* OR disease*)):ti,ab) OR retinopath*:ti,ab OR nephropath*:ti,ab OR neuropath*:ti,ab) AND (‘dementia’/exp OR ‘alzheimer disease’/exp OR ‘vascular dementia’/exp OR dement*:ti,ab OR ((cognitive NEXT/1 (decline OR impairment OR dysfunction)):ti,ab))	( TITLE-ABS-KEY ( diabet* OR “diabetes mellitus” OR “type 1 diabetes” OR “type 2 diabetes” ) AND TITLE-ABS-KEY ( “microvascular complication*” OR “microvascular disease*” OR microangiopath* OR retinopath* OR nephropath* OR neuropath* OR ( microvascular W/1 complication* ) ) AND TITLE-ABS-KEY ( dement* OR “alzheimer* disease” OR “vascular dementia” OR “cognitive decline” OR “cognitive impairment” OR “cognitive dysfunction” OR ( cognitive W/1 decline ) ) )	#1 MeSH descriptor: [Diabetes Mellitus] explode all trees #2 MeSH descriptor: [Diabetes Mellitus, Type 1] explode all trees #3 MeSH descriptor: [Diabetes Mellitus, Type 2] explode all trees #4 diabet*:ti,ab,kw #5 #1 OR #2 OR #3 OR #4 #6 MeSH descriptor: [Diabetic Retinopathy] explode all trees #7 MeSH descriptor: [Diabetic Nephropathies] explode all trees #8 MeSH descriptor: [Diabetic Neuropathies] explode all trees #9 MeSH descriptor: [Microangiopathy] explode all trees #10 (microvascular near/1 complication*):ti,ab,kw #11 (microvascular near/1 disease*):ti,ab,kw #12 retinopath*:ti,ab,kw #13 nephropath*:ti,ab,kw #14 neuropath*:ti,ab,kw #15 #6 OR #7 OR #8 OR #9 OR #10 OR #11 OR #12 OR #13 OR #14 #16 MeSH descriptor: [Dementia] explode all trees #17 MeSH descriptor: [Alzheimer Disease] explode all trees #18 MeSH descriptor: [Vascular Dementia] explode all trees #19 dement*:ti,ab,kw #20 (cognitive near/1 decline):ti,ab,kw #21 (cognitive near/1 impairment):ti,ab,kw #22 (cognitive near/1 dysfunction):ti,ab,kw #23 #16 OR #17 OR #18 OR #19 OR #20 OR #21 OR #22 #24 #5 AND #15 AND #23
